# A retrospective analysis of small molecule targeting inhibitor usage for lung cancer among outpatients in six major regions of China (2016–2022)

**DOI:** 10.3389/fphar.2025.1700389

**Published:** 2025-11-07

**Authors:** Bo Chen, Chen Wang, Li-Ying Chen, Yan Hu, Yu-Zhen Wang, Liu-Cheng Li, Kai-Li Mao, Qiu Jiang

**Affiliations:** 1 Department of Pharmacy, Sir Run Run Shaw Hospital, School of Medicine, Zhejiang University, Hangzhou, Zhejiang, China; 2 Department of Pharmacy, The Quzhou Affiliated Hospital of Wenzhou Medical University, Quzhou People’s Hospital, Quzhou, Zhejiang, China

**Keywords:** lung cancer, small molecule targeting inhibitors, outpatient prescription trends, pharmacoeconomics, targeted therapy

## Abstract

**Objective:**

This study aimed to evaluate national trends in amounts, prescription volumes, and the rationality of drug use in pharmacoeconomics associated with small molecule targeting inhibitors for lung cancer treatment among adult patients in China from 2016 to 2022.

**Methods:**

Prescription data for outpatients diagnosed with lung cancer were extracted from the Hospital Prescription Analysis Cooperative Project database, covering hospitals across six major regions of 77 hospitals. Annual trends in prescription volumes and associated expenditures were analyzed. Additionally, pharmacoeconomic indicators related to small molecule targeting inhibitors were assessed to evaluate cost-effectiveness and utilization patterns. Demographic profiles, regional sources of patients, and classification of small molecule targeting inhibitors were examined.

**Results:**

Prescription volumes and amounts for small molecule targeting inhibitors in lung cancer treatment have increased annually. Since 2020, expenditure levels have stabilized. Furthermore, their use is supported by pharmacoeconomic evidence indicating rational and efficient medication utilization. There was a statistically significant increase in total prescriptions (P_1_ < 0.001) and overall medication expenditures (P_2_ < 0.001).

**Conclusion:**

Between 2016 and 2022, the prescription volume of small molecule targeted inhibitors for lung cancer rose yearly, showing their expanding clinical use. Since 2020, despite continued growth in prescriptions, drug costs have stabilized or slightly decreased. This reflects that China’s medical insurance negotiation and centralized procurement policies have effectively reduced patients’ economic burden without limiting their access to these inhibitors. Pharmacoeconomic indicators also confirm that the use of these drugs has been both reasonable and efficient, allowing for increased drug utilization while reducing patients’ financial strain.

## 1 Introduction

Lung cancer remains the leading malignancy in both incidence and mortality in China. Histologically, it is classified into non-small cell lung cancer (NSCLC) and small cell lung cancer (SCLC), with NSCLC accounting for approximately 80–85% of all cases (Yang et al., 2022; Tang et al., 2025; Lahiri et al., 2023; Shiba-Ishii et al., 2024).

In the treatment of lung cancer, surgical resection is the main treatment approach for early-stage non-small cell lung cancer (NSCLC). However, for some patients with advanced or recurrent disease, targeted therapy plays a significant role in prolonging progression-free survival (PFS) and improving quality of life. Otherwise, the use of targeted drugs before surgery can reduce tumor volume and increase the rate of surgical resection while their use after surgery can lower the risk of recurrence. Surgical indications are usually based on factors such as the stage of the tumor and the overall health of the patient. However, the advent of targeted therapy has provided new treatment options for some patients who were previously not suitable for surgery (Abu-Talib and Brown, 2019; Pezzuto et al., 2015).

Recent advances in cell biology have propelled targeted therapy for NSCLC into a new era. The identification of novel drug targets has introduced new avenues for therapeutic intervention, including EGFR inhibitors, anti-vascular endothelial growth factor (VEGF)/VEGF receptor (VEGFR) monoclonal antibodies, and multi-target agents. Currently, the development of highly selective, low-toxicity, and efficacious anti-NSCLC targeted drugs is a central focus of research. Among these, EGFR is the most frequently targeted mutation in lung cancer therapy (Shiba-Ishii et al., 2024). In a subset of NSCLC patients, mutations in the ALK gene are observed (Goto et al., 2023). Furthermore, during EGFR-targeted therapy, many patients develop resistance due to mutations at the EGFR binding site or activation of bypass signaling pathways. One key bypass mechanism involves the activation of ALK pathways, which significantly contribute to tumor progression and drug resistance. As a result, ALK has become another critical target in the treatment landscape of lung cancer. As of 2022, the small molecule targeting inhibitors approved by China Food and Drug Administration (CFDA) available for lung cancer treatment in China are epidermal growth factor receptor (EGFR) inhibitors including vascular endothelial growth factor (VEGFR) and anaplastic lymphoma kinase (ALK) inhibitors.

This work aimed to assess the usage trends, outpatient volume, drug economics-related indicators, and rationality of small molecule targeted inhibitors for lung cancer treatment among outpatients in 77 hospitals across six major regions in China from 2016 to 2022. Otherwise,it provides reference data for government decision-making departments about medical insurance decisions and promoted rational drug use to clinicians.

## 2 Methods

### 2.1 Study design

This work was conducted as a cross-sectional analysis based on prescription data collected from hospital outpatient records.

### 2.2 Data source and study sample

Prescription data were obtained from the Hospital Prescription Analysis Cooperative Project database, which has been widely used in our previous research and in numerous epidemiological studies across China. The database includes outpatient prescription records from participating hospitals, covering 40 randomly selected days each year. To be included in the study, prescriptions had to meet the following criteria: (i) Contain keywords such as “tinib”; (ii) Be issued to patients diagnosed with lung cancer (regardless of diagnostic criteria, cancer subtype, or disease severity); (iii) Include at least one medication related to lung cancer treatment, whether an initial or renewal prescription, issued between 1 January 2016, and 30 June 2022; (iv) Originate from hospitals located in Beijing, Chengdu, Guangzhou, Hangzhou, Shanghai, or Zhengzhou, which continuously participated in the project throughout the study period. For time reference, the first half of each year is denoted as “s” and the second half as “x” (e.g., “2016s” refers to January–June 2016, and “2016x” refers to July–December 2016). From each qualifying prescription, the following data were extracted: prescription code, patient sex and age, year issued, hospital location, hospital code, diagnosis, generic drug names, and the cost of each medication. As this work was a retrospective study, it not involved specific patient names or other personal information; only codes were used instead. According to the relevant regulations of the ethics committee of our institution, no ethical review was required. This retrospective analysis was conducted in accordance with the ethical guidelines of the World Medical Association and the Declaration of Helsinki. A total of 96,066 outpatient cases were initially identified. Final inclusion was determined after applying the aforementioned inclusion and exclusion criteria.

### 2.3 Assessment of medicine use

Medicine usage was evaluated based on the number of prescriptions, regardless of whether they were newly issued or refills. The total cost was calculated in Chinese Yuan (CNY) by summing the prices of all analyzed medicine. Trends in annual prescription counts and associated costs were analyzed and further stratified by sex, age group, medicine class, and specific drug names.

Prescriptions were categorized according to medications used for lung cancer treatment and any accompanying conditions. “Medicine usage was assessed by the number of prescriptions, regardless of whether they were newly issued or refills. Every prescription meant one outpatient visit. The total cost was calculated in Chinese Yuan (CNY) by summing the prices of all included medications. Trends in annual prescription counts and medication costs were analyzed and further stratified by sex, age, medicine class, and specific drug. Prescriptions were categorized according to the medications used for lung cancer treatment and accompanying conditions. The drugs included epidermal growth factor receptor-tyrosine kinase inhibitors (EGFR-TKIs) such as Afatinib, Gefitinib, Almonertinib, Anlotinib (also classified under VEGFR), Osimertinib, Dacomitinib, Erlotinib, and Furmonertinib, as well as anaplastic lymphoma kinase-tyrosine kinase inhibitors (ALK-TKIs), including Ensartinib, Alectinib, Crizotinib, and Ceritinib.”

### 2.4 Data analysis

Statistical significance was set at P < 0.05. All prescription data were processed using Microsoft Office Excel, Version 2016 (Microsoft Corp., Redmond, WA, United States). Figures were created using GraphPad Prism, Version 9. All statistical analyses were conducted using R software, Version 4.3.2 (http://www.R-project.org).

## 3 Results

### 3.1 Demographic characteristics of outpatients using small molecule targeting inhibitors and trends in overall usage

A total of 96,066 outpatient visits involving small molecule targeting inhibitors for lung cancer treatment were recorded across 77 hospitals in six major Chinese cities. The demographic characteristics of the overall study population, along with stratified data by age group, city, hospital level, and sex from 2018 to 2022, are presented in Table 1.

**TABLE 1:** Demographic composition of patients by gender, age group, city and hospital level.

	2016s	2016x	2017s	2017x	2018s	2018x	2019s	2019x	2020s	2020x	2021s	2021x	2022s	P_1_	p_2_
city															
Beijing	227	254	389	502	739	1,005	1,472	1771	1,437	1,374	1716	1849	1959	*P*_*1*_ *< 0.001*	*P*_*2*_ *< 0.001*
Chengdu	0	0	0	46	219	701	1,241	1,440	1,255	1,096	886	1,028	1,022	*P*_*1*_ *= 0.014*	*P*_*2*_ *< 0.001*
Guangzhou	461	368	522	594	1,133	1,576	2,977	3,455	3,712	3,845	4,386	4,428	5,014	*P*_*1*_ *< 0.001*	*P*_*2*_ *< 0.001*
Hangzhou	479	502	455	454	690	792	1,325	1,597	1878	1813	2,143	2,300	3,081	*P*_*1*_ *< 0.001*	*P*_*2*_ *= 0.002*
Shanghai	207	172	225	463	453	525	955	1,198	1,420	1,494	1,518	1,659	903	*P*_*1*_ *< 0.001*	*P*_*2*_ *< 0.001*
Zhengzhou	142	257	484	621	701	686	992	1,196	1,232	1,188	1,252	1,266	1,249	*P*_*1*_ *< 0.001*	*P*_*2*_ *< 0.001*
Hospital level															
Second-class Hospital	0	0	0	0	0	0	2	2	12	8	20	9	18	*P*_*1*_ *< 0.001*	*P*_*2*_ *< 0.001*
tertiary hospitals	1,516	1,553	2075	2,680	3,935	5,285	8,960	10,655	10,922	10,802	11,881	12,521	13,210	*P*_*1*_ *< 0.001*	*P*_*2*_ *< 0.001*
sex															
male	647	654	852	1,127	1,576	2081	3,831	4,549	4,594	4,497	5,054	5,215	5,458	*P*_*1*_ *< 0.001*	*P*_*2*_ *= 0.735*
female	869	899	1,223	1,553	2,359	3,204	5,131	6,108	6,340	6,313	6,847	7,315	7,770	*P*_*1*_ *< 0.001*	*P*_*2*_ *= 0.735*
age															
<30	2	122	6	4	4	8	33	40	51	60	45	50	75	*P*_*1*_ *= 0.01*	*P*_*2*_ *< 0.001*
30–39	26	27	56	62	71	92	218	277	290	314	335	376	335	*P*_*1*_ *< 0.001*	*P*_*2*_ *< 0.001*
40–49	162	145	238	275	341	459	838	1,006	934	1,069	1,070	1,207	1,140	*P*_*1*_ *< 0.001*	*P*_*2*_ *< 0.001*
50–59	324	323	459	630	981	1,206	2,166	2,666	2,787	2,754	3,002	3,216	3,394	*P*_*1*_ *< 0.001*	*P*_*2*_ *< 0.001*
60–69	519	495	715	978	1,346	1903	3,168	3,664	3,743	3,590	3,920	4,160	4,450	*P*_*1*_ *< 0.001*	*P*_*2*_ *< 0.001*
≥70	483	441	601	731	1,192	1,617	2,539	3,004	3,129	3,023	3,529	3,521	3,834	*P*_*1*_ *< 0.001*	*P*_*2*_ *= 0.378*

*P*_*1*_ refers to the p-value for the trend in the number of prescriptions, assessed using the Mann-Kendall trend test. *P*_*2*_ refers to the p-value for the trend in the proportion of prescriptions, assessed using the Cochran-Armitage trend test. When the data volume is fewer than three points, the calculation of *P*_*1*_ and *P*_*2*_ is not feasible.

Prescription volume increased significantly over time for both male and female patients, with strong statistical significance observed in both groups (*P*_*1*_ *<* 0.001). However, the proportion of gender remained unchanged. When stratified by age group, all age categories showed statistically significant trends in prescription volume (*P*_*1*_ < 0.001, *P*_*2*_ *<* 0.001), and for patients under 30 years (*P*_*1*_ = 0.010, *P*_*2*_ *<* 0.001), except for those aged 70 years or older (*P*_*1*_ < 0.001, *P*_*2*_ = 0.378), as shown in Table 1. Across the six cities, prescription volumes demonstrated significant temporal variation. In four cities—Beijing, Shanghai, Guangzhou, and Zhengzhou—both trend tests yielded highly significant results (*P*_*1*_ < 0.001, *P*_*2*_ *<* 0.001). In Chengdu and Hangzhou, significant but slightly weaker trends were observed (*P*_*1*_ = 0.014 for Chengdu, *P*_*2*_ = 0.002 for Hangzhou). Regarding hospital levels, both secondary and tertiary institutions exhibited statistically significant increases in prescription volume over time (*P*_*1*_ < 0.001, *P*_*2*_ *<* 0.001), as detailed in Table 1.

### 3.2 Prescriptions and expenditures of small molecule targeting inhibitors for lung cancer

**FIGURE 1:**
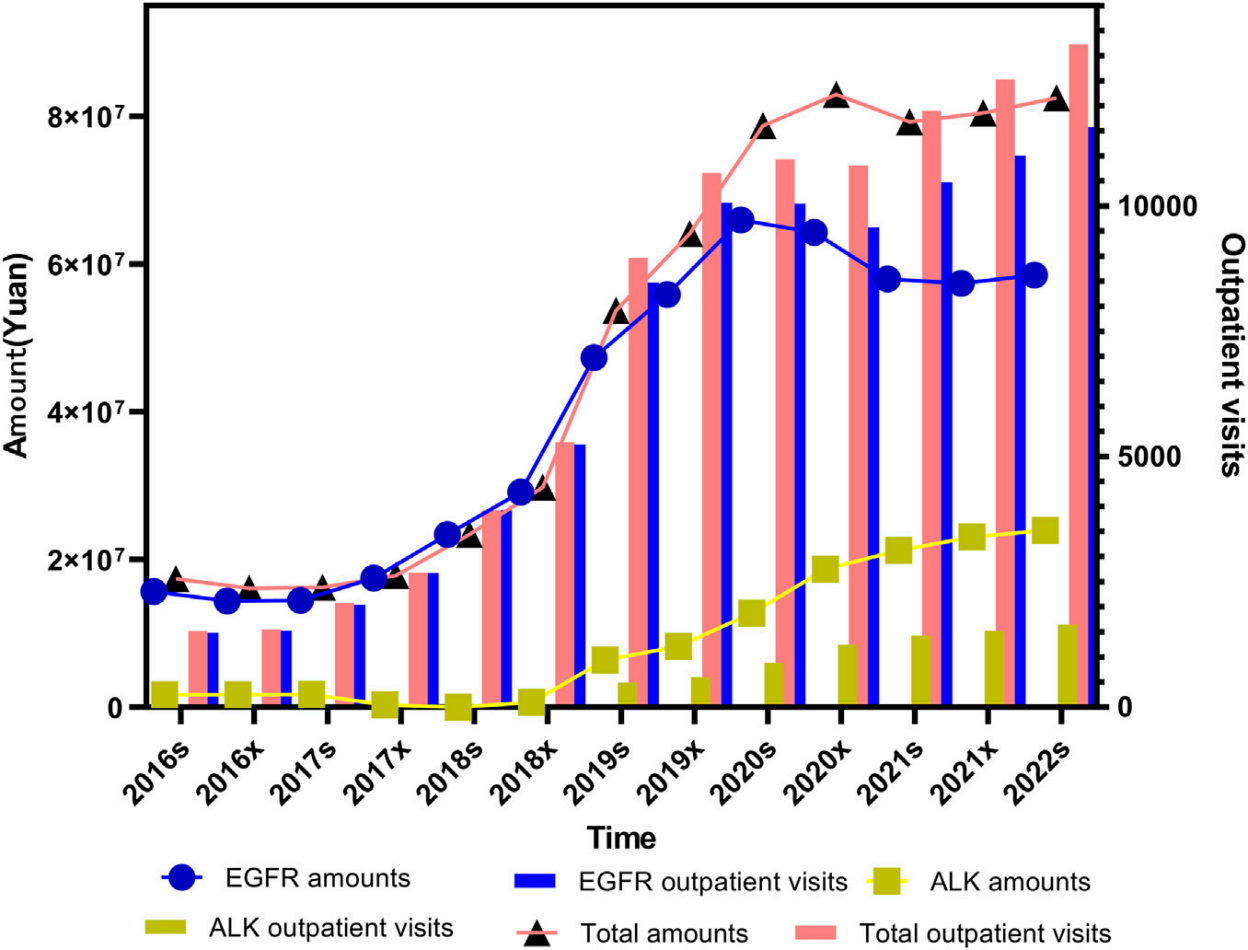
Trends in outpatient visits and total prescription expenditures for small molecule targeting inhibitors targeting lung cancer from 2016s to 2022s.

As illustrated in Figure 1, “outpatient visits” are represented as a bar chart, with values plotted against the right Y-axis. The remaining line graphs reflect prescription costs, corresponding to the left Y-axis. Both total outpatient visits and expenditures demonstrated statistically significant increasing trends over time (*P <* 0.001, Mann-Kendall trend test).

For ALK inhibitors, expenditure levels and outpatients remained relatively low prior to the second half of 2018. This was primarily because Crizotinib was the only approved ALK inhibitor available on the market at that time. Following the market introduction of Ceritinib, Alectinib, and Ensartinib in the latter half of 2018, a noticeable increase in ALK-related expenditure and outpatient visits was observed in each subsequent half-year period. EGFR inhibitor prescriptions, in contrast, showed a sharp upward trend until the first half of 2020. After that point, the growth began to plateau and eventually showed a mild decline. When comparing the trends in total outpatient visits and total expenditures for small molecule targeting inhibitors over the study period, it is evident that reduction of EGFR inhibitors in cost have declined over time. This reduction in cost is likely attributable to the inclusion of these medicines in Chinese medical insurance negotiation, which has prompted significant price adjustments. Additionally, the introduction of generic alternatives through centralized procurement initiatives or the expiration of patent protection has also played a key role in driving down prices.

### 3.3 Prescriptions and expenditures of EGFR inhibitors for lung cancer treatment

The EGFR inhibitors used in lung cancer treatment are classified into three generations. The first-generation EGFR inhibitors include Gefitinib, Icotinib, and Erlotinib. These drugs affect both mutant and wild-type EGFR, although their selectivity is limited. The second-generation inhibitors, such as Afatinib and Dacomitinib, were developed to improve selectivity for EGFR mutations and reduce effects on wild-type receptors, but they offer no substantial advantages in terms of efficacy or side effects compared to the first generation. The third-generation EGFR inhibitors, including Almonertinib, Osimertinib, and Fumituximab, are designed to overcome the T790M resistance mutation and demonstrate improved selectivity for mutant EGFR while reducing off-target effects on wild-type receptors.

For the first-generation EGFR inhibitors, the trends in prescription volume and cost were not entirely consistent (*P*_*1*_ = 0.033, *P*_*2*_ *<* 0.001).

The prescription volume and expenditure for second-generation EGFR inhibitors followed a similar pattern. Both remained relatively low before the second half of 2018 (2018x), gradually increased until the first half of 2021 (2021s), and then stabilized through the first half of 2022 (2022s). These trends were statistically significant for both metrics (*P*_*1*_ < 0.001, *P*_*2*_ *<* 0.001). The reason for the outpatient visits and expenditure of the second-generation EGFR were lower than those of the first and third generations was that the clinical application of second-generation irreversible inhibitor afatinib was limited due to its poor therapeutic window and third-generation EGFR TKIs was developed for effective management of T790M-associated resistance while sparing wild-type EGFR (Chen et al., 2018).

**FIGURE 2:**
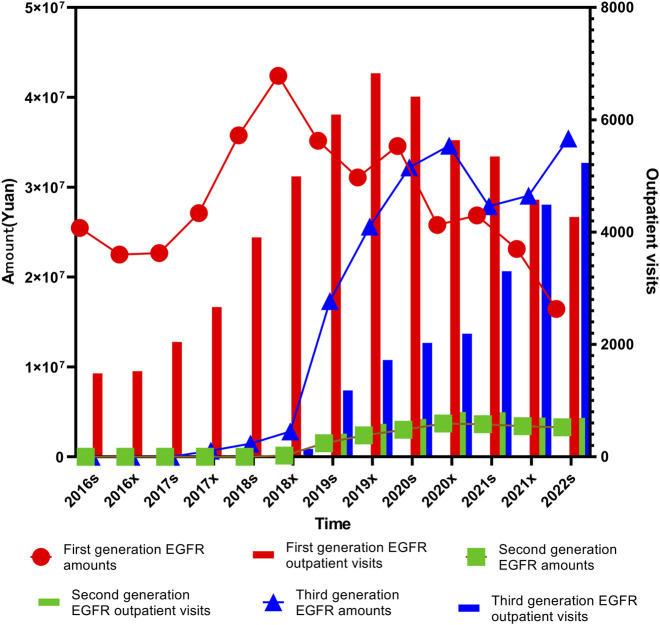
Trends in prescription expenditures and outpatient visits of first-, second-, and third-generation EGFR inhibitors from 2016s to 2022s.

The third-generation EGFR inhibitors demonstrated a consistently increasing trend in prescription volume across the study period (*P*_*1*_ < 0.001, *P*_*2*_ *<* 0.001). However, while total expenditures also rose significantly between 2016 and 2020 (*P*_*1*_ < 0.001, *P*_*2*_ *<* 0.001), the rate of increase slowed after 2020. This likely reflects the impact of price reductions resulting from inclusion in the national medical insurance directory and subsequent pricing negotiations, as shown in Figure 2.

In order to further clarify the specific reasons for the differences in outpatient visits and expenditure of first-, second-, and third-generation EGFR inhibitors, we specifically analyzed the medicines of each generation of medication. Gefitinib, launched in February 2005, was among the earliest EGFR inhibitors approved for clinical use in China and saw a significant price reduction following its inclusion in the national centralized procurement program in 2019. Erlotinib, also introduced in 2005, remains in clinical use despite the emergence of newer-generation alternatives. Anlotinib entered the market in May 2018, followed by Dacomitinib in September 2018 and Afatinib in February 2017. Icotinib, which was launched in June 2011, was added to centralized the centralized procurement list in 2021. More recently, Furmonertinib was approved in March 2021. Osimertinib, introduced in March 2017, has since become widely adopted, particularly after being endorsed by the China Lung Cancer Treatment Guidelines10] as a first-line treatment. It is used not only for patients with acquired T790M resistance mutations but also for those with locally advanced NSCLC harboring EGFR exon 19 deletions or exon 21 (L858R) substitutions (Lamb, 2021). Almonertinib, another third-generation agent, was approved in April 2020 and has gained traction in clinical settings for similar indications.

Statistical analysis of the expenditure trends associated with these drugs revealed varying degrees of significance. Afatinib showed a modest but notable increase in total cost over time (*P*_*1*_ = 0.064), alongside a highly significant increase in its proportional share (*P*_*2*_ *<* 0.001). Almonertinib showed statistically significant growth in both expenditure (P_1_ = 0.028) and proportion (*P*_*2*_ *<* 0.001). Icotinib, Osimertinib, and Erlotinib exhibited strong statistical significance across both metrics (*P*_*1*_ *and P*_*2*_ *<* 0.001), while Anlotinib’s total cost trend did not reach statistical significance (*P*_*1*_ = 0.108) despite a notable increase in proportional usage (*P*_*2*_ *<* 0.001). Gefitinib demonstrated only a borderline significant change in total cost (*P*_*1*_ = 0.127), though its proportional usage rose significantly (*P*_*2*_ *<* 0.001).

**FIGURE 3:**
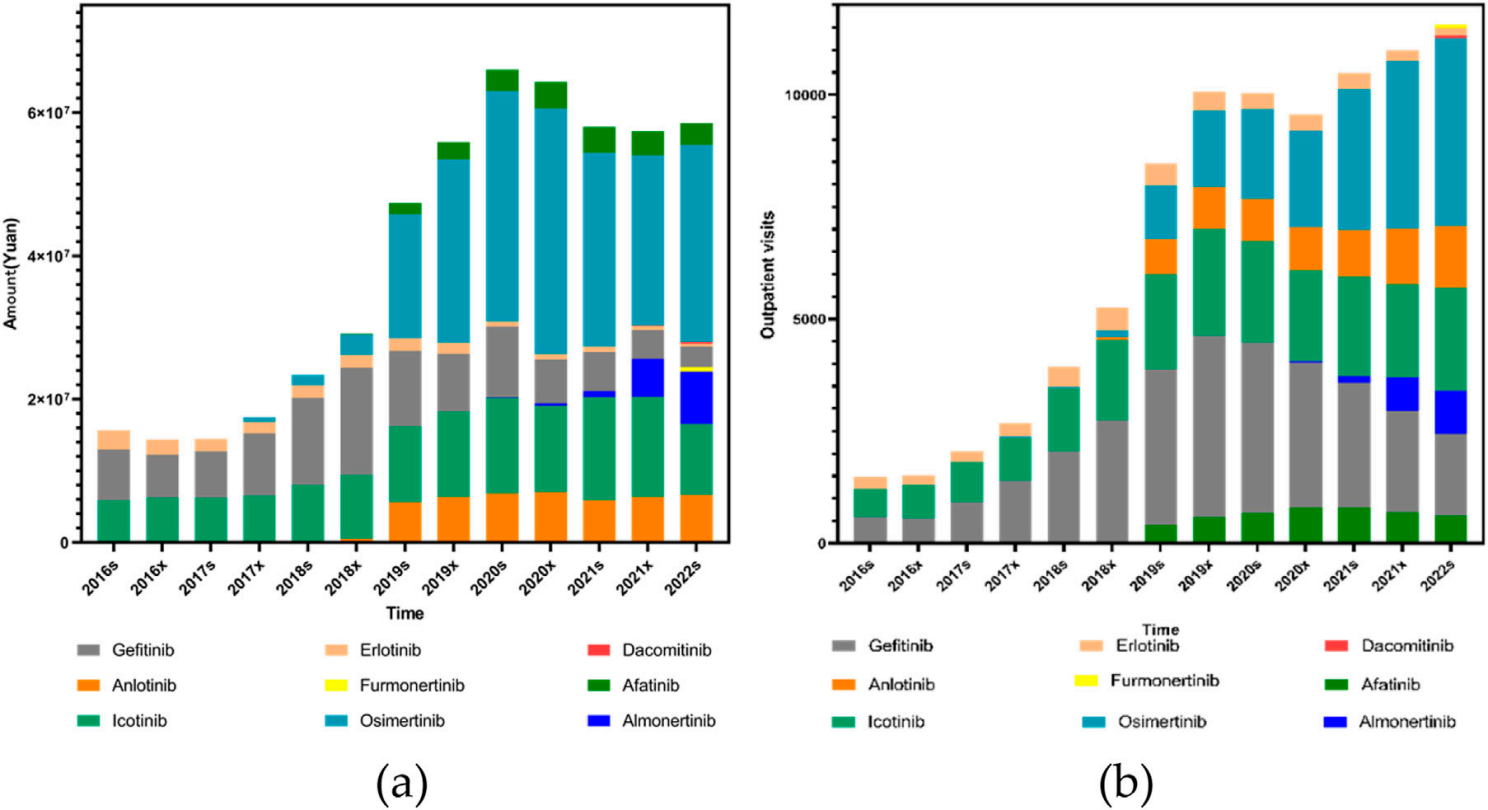
Trends in the use of EGFR inhibitors (including Anlotinib as a VEGFR-targeted agent) from 2016s to 2022s: **(a)** Total prescription expenditures; **(b)** Outpatient outpatient visits.

Analysis of outpatient visit volumes further supported these trends. Afatinib, Icotinib, Osimertinib, Anlotinib, and Almonertinib (also referred to as Almonertinib in certain data sources) all showed statistically significant increases in both volume and proportional visit share over time (*P*_*1*_ *and P*_*2*_ *<* 0.001). Erlotinib, in contrast, exhibited no significant trend in visit volume (*P*_*1*_ = 0.760), although its proportional increase remained significant (*P*_*2*_ *<* 0.001). Gefitinib presented a borderline significant increase in visit volume (*P*_*1*_ = 0.059) and a statistically significant rise in its proportional share (*P*_*2*_ *<* 0.001), as shown in Figure 3.

### 3.4 Prescriptions and expenditures of ALK inhibitors for lung cancer treatment

**FIGURE 4:**
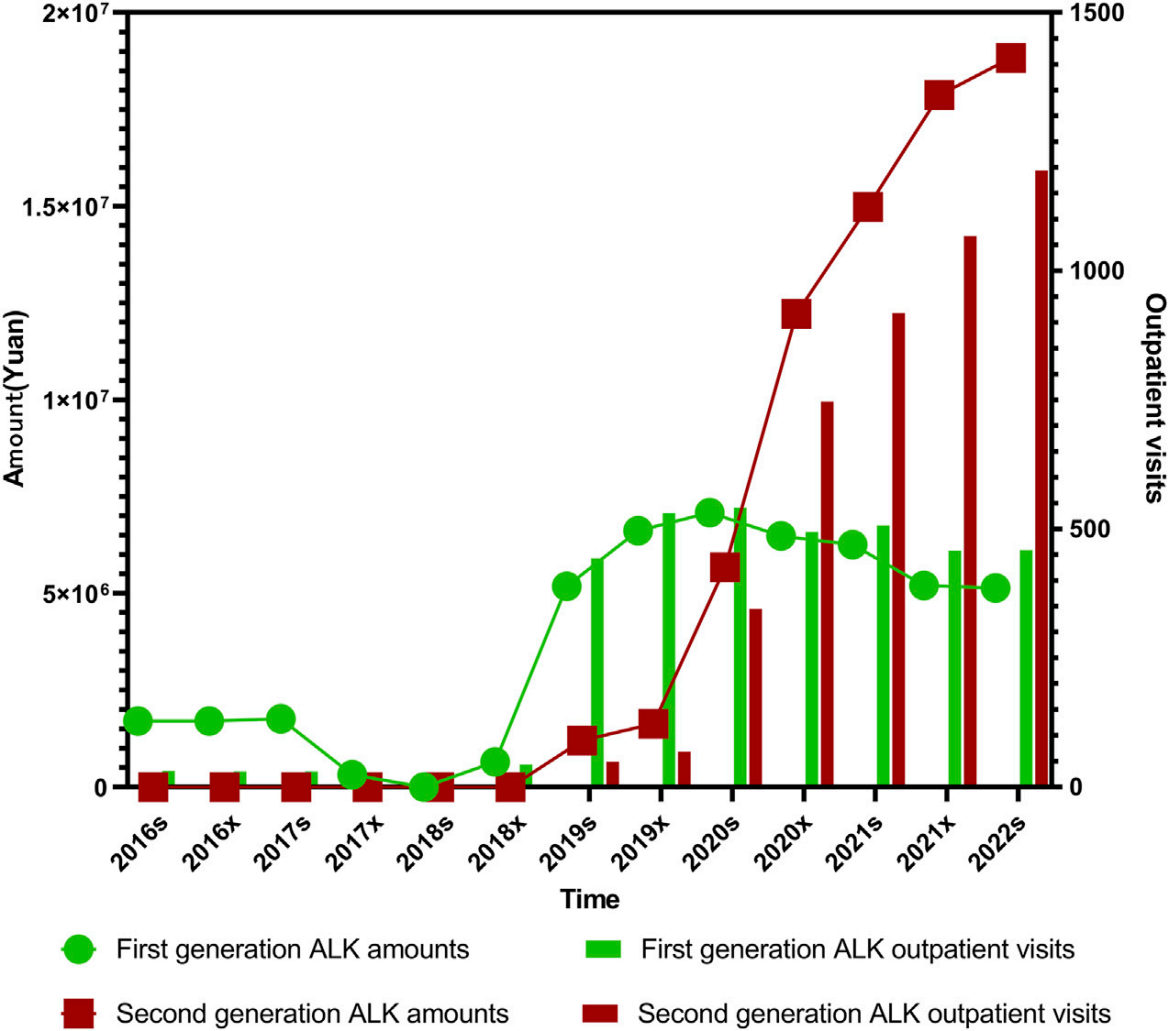
Trends in prescription expenditures and outpatient visits of first- and second-generation ALK inhibitors from 2016s to 2022s.

The overall trends in expenditures and outpatient visits for first- and second-generation ALK (anaplastic lymphoma kinase) inhibitors followed a broadly similar pattern throughout the study period. From the first half of 2016 (2016s) to the second half of 2018 (2018x), both prescription volume and expenditure for first-generation ALK inhibitors remained at relatively low levels. A sharp increase was observed from 2018x to the first half of 2020 (2020s), followed by a plateau and a gradual decline extending through the first half of 2022 (2022s). Statistical analysis showed that while the expenditure trend for first-generation ALK inhibitors was not significant (*P*_*1*_ = 0.112), the change in proportional use was significant (*P*_*2*_ *<* 0.001). In contrast, second-generation ALK inhibitors demonstrated statistically significant upward trends in both expenditure (*P*_*1*_ *<* 0.001) and proportion (*P*_*2*_ *<* 0.001). Similarly, outpatient visits for first-generation inhibitors showed a significant increase (*P*_*1*_ = 0.0038, *P*_*2*_ *<* 0.001), with second-generation agents also showing highly significant growth (*P*_*1*_ < 0.001, *P*_*2*_ *<* 0.001), as illustrated in Figure 4.

In order to further clarify the specific reasons for the differences in outpatient visits and expenditure of first-, second- ALK inhibitors, we specifically analyzed the medicines of each generation of medication. Among the ALK-targeted tyrosine kinase inhibitors (TKIs), Enshatinib is approved as monotherapy for patients with locally advanced or metastatic non-small cell lung cancer (NSCLC) harboring ALK-positive mutations. Ceritinib is also indicated for ALK-positive NSCLC, while Alectinib is approved for both advanced disease and as postoperative adjuvant therapy in ALK-positive patients with stage IB to IIA NSCLC. Crizotinib, the first ALK inhibitor introduced, is used for ALK-positive NSCLC and additionally for cases with ROS1-positive mutations.

The trends in both outpatient visits and expenditures across these four ALK inhibitors were generally consistent. Total usage remained low between 2016s and 2018x, followed by a pronounced and sustained increase from 2018s to 2022x. Alectinib, launched in China in August 2018, remained underutilized until 2019s, after which both its outpatient visits and expenditure rose sharply, showing highly significant trends (*P*_*1*_ < 0.001, *P*_*2*_ *<* 0.001 for both). Enshatinib, approved in November 2020, and Ceritinib, launched in May 2018, exhibited gradual but steady increases in usage. For Ceritinib, both outpatient visits and total expenditure showed significant upward trends (*P*_*1*_ < 0.001, *P*_*2*_ *<* 0.001).

Crizotinib usage remained low and stable prior to 2018x. However, from 2018x to 2020s, both its volume and cost rose rapidly before entering a slow decline, maintaining relatively high levels thereafter. Crizotinib’s outpatient visits and expenditures were both statistically significant over the period (*P*_*1*_ = 0.038 *and* 0.033 respectively, *P*_*2*_ *<* 0.001 for both). Among all four ALK inhibitors, Crizotinib and Alectinib together accounted for more than 80% of total outpatient visits and overall drug use, which is closely linked to their early inclusion in China’s national medical insurance system.

**FIGURE 5:**
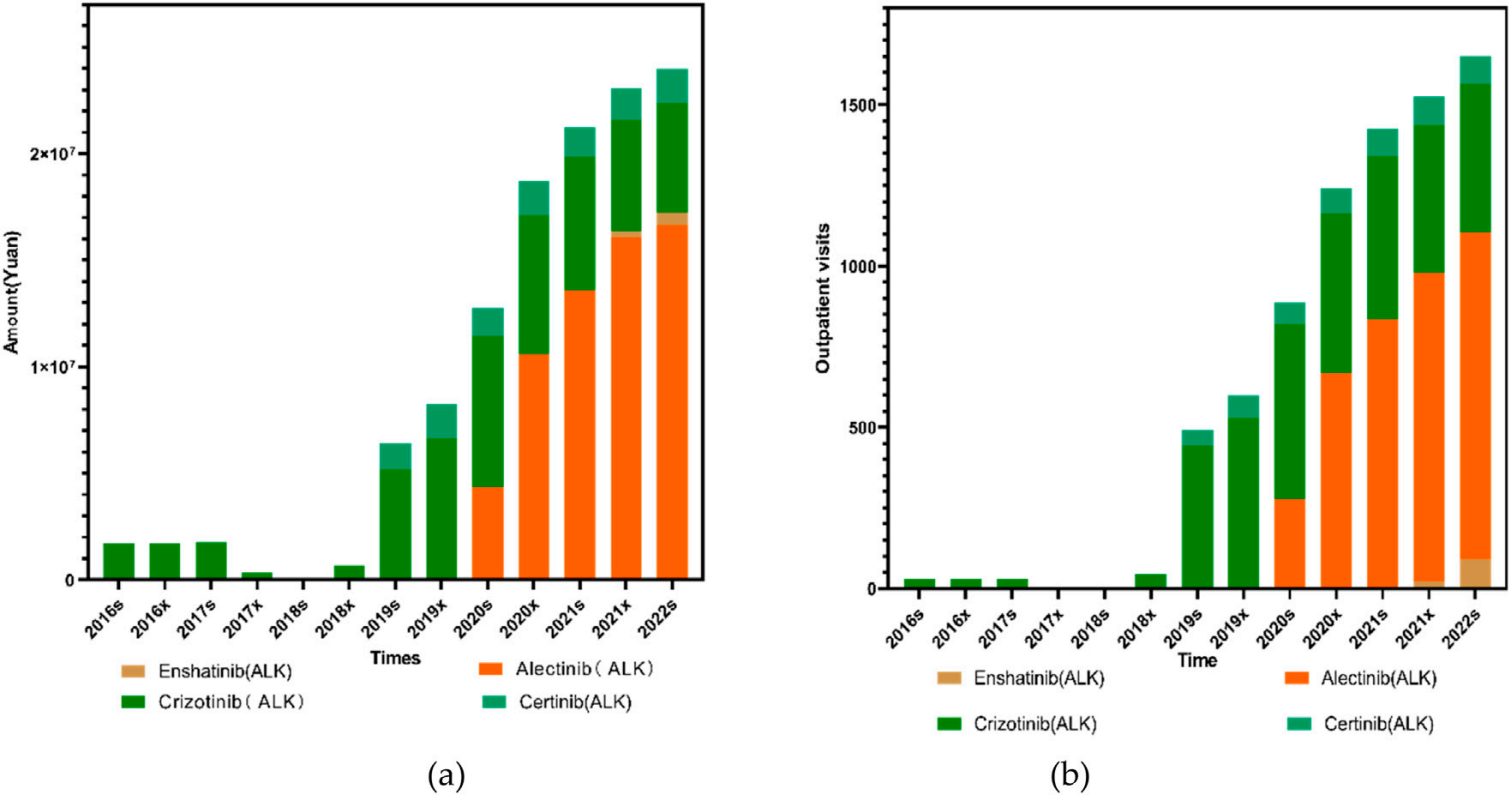
Trends in the use of ALK inhibitors from 2016s to 2022s: **(a)** Total prescription expenditures; **(b)** Outpatient visit volume.

Clinically, second-generation ALK inhibitors have demonstrated clear advantages over their first-generation counterparts, offering improved progression-free survival and reduced resistance. They are also associated with fewer adverse effects, contributing to better patient quality of life and long-term outcomes, as illustrated in Figure 5.

### 3.5 Pharmacoeconomics and rationality of small molecule inhibitor use in lung cancer treatment

The Defined Daily Dose (DDD) values for small molecule targeting inhibitors used in lung cancer treatment were primarily obtained from the World Health Organization (WHO) database. For medications without an officially published DDD on the WHO website, values were determined based on the dosage recommendations in official drug labels and the clinical guidelines issued in China. The DDD values applied in this study were as follows: Afatinib, 40 mg; Furmonertinib, 80 mg; Almonertinib, 110 mg; Icotinib, 375 mg; Osimertinib, 80 mg; Dacomitinib, 45 mg; Erlotinib, 0.15 g; Gefitinib, 0.25 g; Alectinib, 1.2 g; Ensartinib, 225 mg; Crizotinib, 0.5 g; and Ceritinib, 0.45 g.

The total dosage divided by the Defined Daily Doses (DDDs) revealed a clear upward trend for nearly all small molecule targeting inhibitors targeting lung cancer, with the exception of Gefitinib, Erlotinib, and Afatinib—representatives of the earlier generations of EGFR-targeted therapies. The increasing DDDs suggest that clinical adoption of these therapies is expanding, reflecting both broader physician preference and greater patient accessibility.

In contrast, the Defined Daily Drug Cost (DDDc) values for most small molecule targeting inhibitors showed a consistent downward trend over the study period. This decline in DDDc indicates a reduced economic burden on patients, likely due to Chinese medical insurance negotiations and centralized procurement policies, and increasing availability of generic alternatives.

**FIGURE 6:**
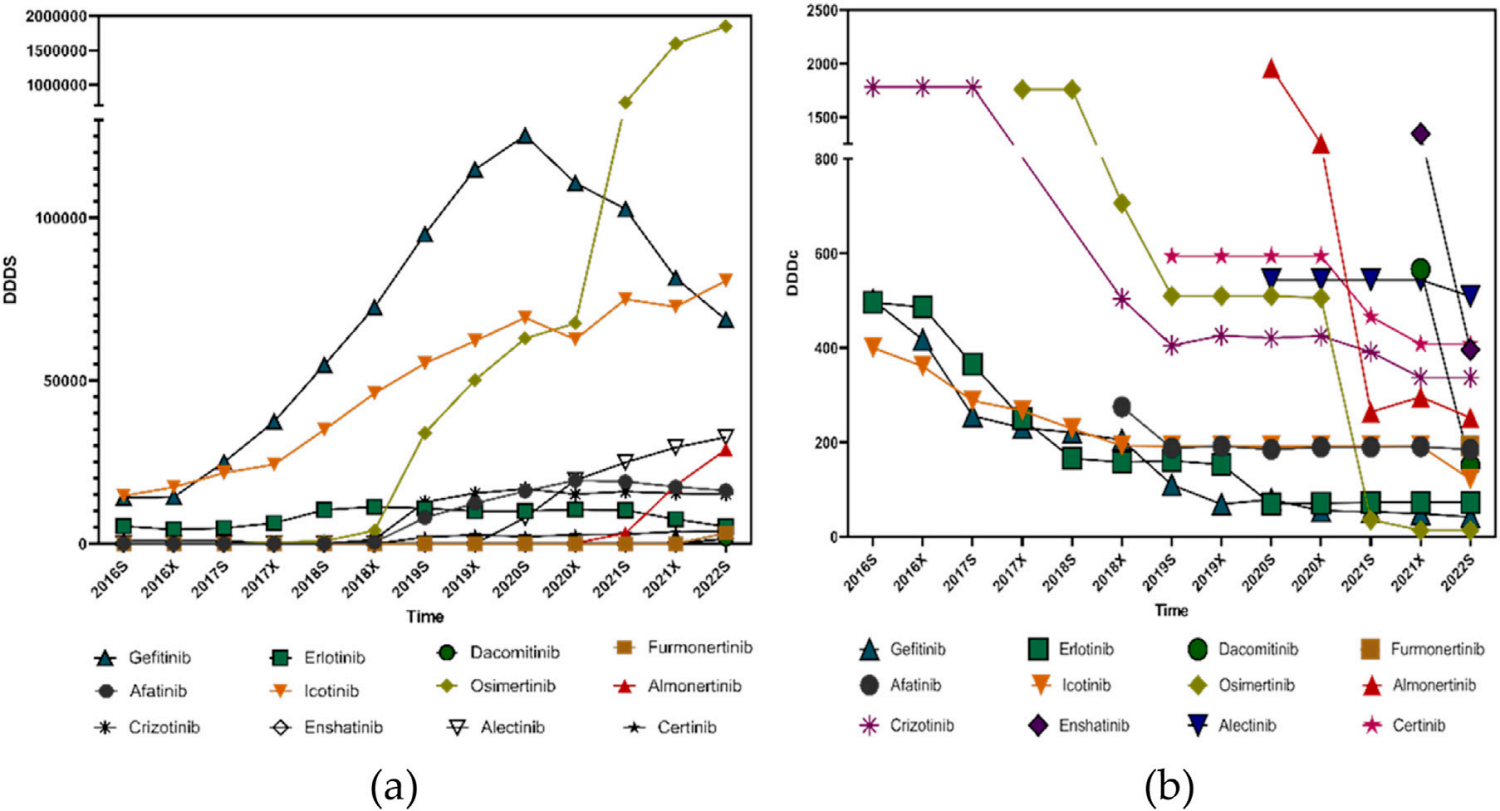
Pharmacoeconomic trends of small molecule targeting inhibitors targeting lung cancer from 2016s to 2022s: **(a)** DDDs; **(b)** DDDc.

Taken together, the simultaneous rise in DDDs and fall in DDDc values strongly support the pharmacoeconomic rationality of small molecule inhibitor use in lung cancer treatment within the Chinese healthcare context. The findings suggest that these therapies are not only increasingly utilized but are also becoming more affordable, enhancing both accessibility and sustainability in clinical practice as illustrated in Figure 6.

## 4 Discussion

From 2016s to 2022x, outpatient visits for small molecule targeting inhibitors in the treatment of lung cancer have increased steadily, with the exception of a slight decline observed in 2020x. However, this growth in prescription volume did not correspond to a continued rise in total expenditure. Prior to 2020x, the total prescription cost increased alongside the rise in outpatient visits. After 2020x, however, a gradual decline in total drug expenditure was observed despite continued growth in patient volume. This trend suggests that the inclusion of several small molecule targeting inhibitors in Chinese medical insurance negotiations framework, along with centralized procurement policies, has played a significant role in reducing drug prices and alleviating the economic burden on patients.

Existing research focused on OS (overall survival) in treatment of lung cancer (Griesinger et al., 2024),and some research were focused on immunotherapy (Fletcher et al., 2024; Wu et al., 2023; Yang et al., 2020; Dai et al., 2025). But targeted therapeutic medicines still played a important role in lung cancer (Chen et al., 2022). The existing studies did not investigate the prescription trends and economic aspects of specific small molecule targeting inhibitors. This work was the first time to investigate the use of small molecule targeting inhibitors for lung cancer in six major regions of China, and also assessed the rationality of the use of these drugs.

This study analyzed prescribing trends of EGFR and ALK-small molecule targeting inhibitors using a large anonymized prescription database from hospitals in six major Chinese cities. Overall, EGFR inhibitors have dominated the prescription volume due to the higher prevalence of EGFR mutations in lung cancer. However, while EGFR-related prescriptions increased significantly in the earlier years, a slow decline was observed beginning in 2020a. In contrast, ALK inhibitor prescriptions demonstrated a consistent upward trend throughout the entire study period. Despite this growth, EGFR-targeted therapies still represent the majority of total prescriptions. Regarding individual medicines, Gefitinib was the most expensive EGFR-targeted therapy in use prior to 2019s. From 2019s onward, Osimertinib became the most expensive EGFR inhibitor prescribed. A shift was also observed in terms of usage volume: prior to 2021s (exclusive), Gefitinib accounted for the highest number of outpatient visits, whereas from 2021s (inclusive), Osimertinib took the lead. A similar pattern emerged among ALK inhibitors. Crizotinib had the highest cost and prescription volume before 2020x, but from 2020x onward, Alectinib surpassed it in both expenditure and prescription frequency. These trends reflect changes in clinical practice, availability of newer-generation therapies, and policy-driven cost reductions.

Nevertheless, this study has certain limitations. The prescription data were obtained from hospitals in six major urban areas participating in the Hospital Prescription Analysis Cooperative Project. While these cities are representative of developed healthcare settings in China, they may not fully reflect national prescribing patterns, particularly in rural or less developed regions. Moreover, the data represent prescriptions issued but do not confirm whether patients adhered to or completed the prescribed therapies.

Another limitation lies in the nature of the sampling process. The data were collected based on randomized days throughout the year, which may have resulted in the omission of some prescriptions or drug combinations. Additionally, the dataset did not allow for tracking continuous medication changes in individual patients over time. Therefore, it was not possible to assess treatment duration, sequencing, or switching patterns, which are crucial for real-world effectiveness studies. Given the scale of the study spanning 77 hospitals and 96,066 patient visits in six major areas of China, systematically assessing overall survival, disease progression, and treatment adherence was not feasible. And the data source is the “Hospital Prescription Analysis Cooperative Project database,” which only includes 77 hospitals across six regions of China. This may limit the national representativeness of the findings. Future research should aim to address these limitations by incorporating longitudinal patient-level data and expanding geographic coverage to include a more diverse population and adding relevant content that future clinical practice based on big data for prescription analysis and management. Meanwhile, a nationwide analysis of small molecule inhibitor use in lung cancer, incorporating adherence, treatment response, and outcome data, will be essential to comprehensively evaluate the clinical and pharmacoeconomic impact of these therapies in real-world settings.

## Data Availability

The original contributions presented in the study are included in the article/supplementary material, further inquiries can be directed to the corresponding author.

## Abbreviations

ALK, Anaplastic Lymphoma Kinase; ROS1, ROS Proto-Oncogene 1 (c-ros oncogene 1); VEGF, Vascular Endothelial Growth Factor; TKI, Tyrosine Kinase Inhibitor; DDD, Defined Daily Dose; DDDs, The total dosage divided by the Defined Daily Dose; DDDc, Defined Daily Drug Cost; P_1_, P-value for trend in absolute prescription volume (Mann-Kendall test); P_2_, P-value for trend in prescription proportion (Cochran-Armitage test); CNY, Chinese Yuan; WHO, World Health Organization; SPSS, Statistical Package for the Social Sciences; OS, Overall Survival.
